# Control of Dendritic Spine Morphological and Functional Plasticity by Small GTPases

**DOI:** 10.1155/2016/3025948

**Published:** 2016-02-18

**Authors:** Kevin M. Woolfrey, Deepak P. Srivastava

**Affiliations:** ^1^Department of Pharmacology, University of Colorado School of Medicine, Anschutz Medical Campus, Aurora, CO 80045, USA; ^2^Department of Basic and Clinical Neuroscience, Maurice Wohl Clinical Neuroscience Institute, Institute of Psychiatry, Psychology and Neuroscience, King's College London, London SE5 9RT, UK

## Abstract

Structural plasticity of excitatory synapses is a vital component of neuronal development, synaptic plasticity, and behaviour. Abnormal development or regulation of excitatory synapses has also been strongly implicated in many neurodevelopmental, psychiatric, and neurodegenerative disorders. In the mammalian forebrain, the majority of excitatory synapses are located on dendritic spines, specialized dendritic protrusions that are enriched in actin. Research over recent years has begun to unravel the complexities involved in the regulation of dendritic spine structure. The small GTPase family of proteins have emerged as key regulators of structural plasticity, linking extracellular signals with the modulation of dendritic spines, which potentially underlies their ability to influence cognition. Here we review a number of studies that examine how small GTPases are activated and regulated in neurons and furthermore how they can impact actin dynamics, and thus dendritic spine morphology. Elucidating this signalling process is critical for furthering our understanding of the basic mechanisms by which information is encoded in neural circuits but may also provide insight into novel targets for the development of effective therapies to treat cognitive dysfunction seen in a range of neurological disorders.

## 1. Introduction

**Figure 1:**
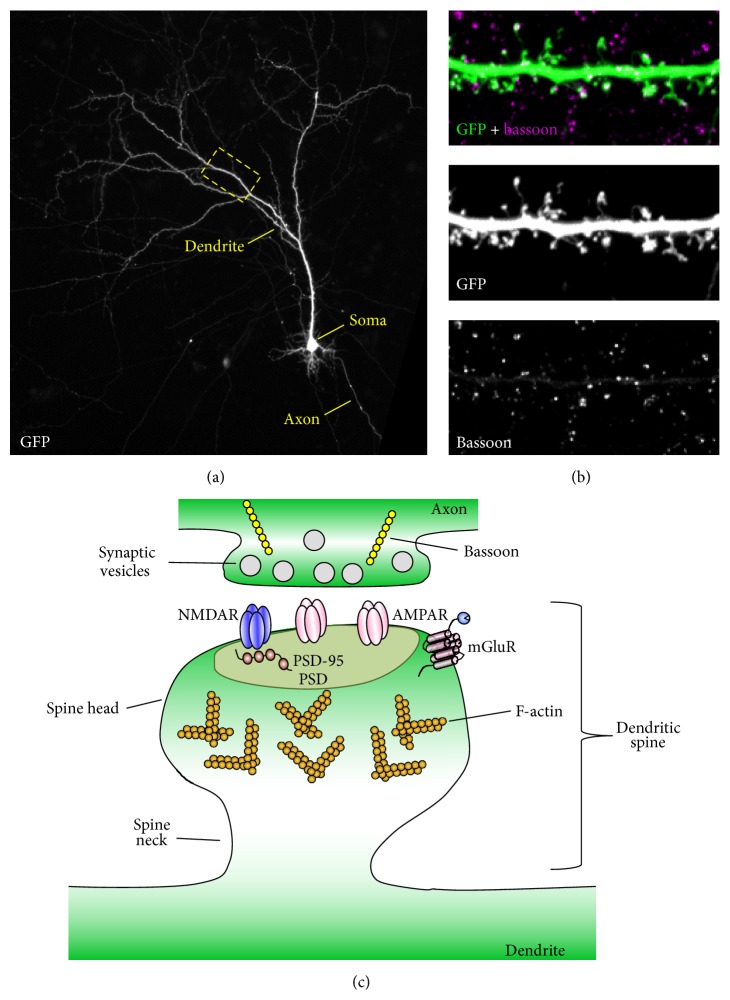
Dendritic spines are small protrusions along dendrites that contain postsynaptic densities. (a) Example of a cortical neuron expressing green fluorescent protein (GFP). The main dendrite is branched and has dendritic spines along its length. Dashed box indicates area magnified in (b). The neuron's axon is much thinner than the dendrite and has no spines. (b) Magnified region of dendrite of a cortical neuron expressing GFP and stained for the presynaptic protein bassoon. Dendritic spines can clearly be seen protruding from the dendrite, and many spines colocalize with bassoon, suggesting the formation of synaptic connections. In this colour scheme, colocalization is indicated by white. (c) Schematic of a mature dendritic spine making contact with an axon; note the enrichment of glutamate receptors, the scaffold protein PSD-95, and F-actin within the spine head and postsynaptic density (PSD).

Brain function is an emergent property of the connections between neurons. Proper wiring of the brain during development is critical for cognition and memory [1–3], while, conversely, abnormal wiring due to neurological disorder, disease, or brain injury results in dysfunction [4–6]. Understanding how neural circuitry underlies information storage and processing is a fundamental challenge facing modern neuroscience [1, 3]. Though modest inroads into deciphering brain wiring have been made, very little is known about how this wiring contributes to its function. A primary obstacle to progress is the staggering complexity of neural circuits; in mammalian brains, trillions of synapses impinge on billions of neurons. One approach to managing this complexity is to limit focus to synapses of a single neurotransmitter type. Glutamatergic synapses are highly plastic, play essential roles in learning, memory, as well as cognition, and comprise the majority of the connections between pyramidal neurons in the forebrain [7–9]. A defining characteristic of these synapses is that they occur at specialized postsynaptic compartments known as dendritic spines (Figures 1(a)–1(c)). These micron-scale, actin-rich structures garnish the dendritic arbour and typically consist of a spine neck and a spine head [10, 11]. It is within the spine head that the protein-rich postsynaptic density (PSD) is found (Figure 1(c)). Embedded in the PSD are *N*-Methyl-D-aspartic acid (NMDA) and *α*-amino-3-hydroxy-5-methyl-4-isoxazolepropionic acid (AMPA) type glutamate receptors which mediate excitatory synaptic transmission (Figure 1(c)) [10, 12]. Dendritic spines exhibit both transient and enduring lifetimes, persisting from minutes to years* in vivo* [7, 13]. A myriad of dendritic spine morphologies are observed in the brain and the notion that spine structure is highly correlated with important synaptic properties has become a recurrent theme over the last decade [14, 15]. For example, large dendritic spines are likely to feature large PSDs and make strong connections, while small dendritic spines are indicative of weak connections and may be highly plastic [16]. Accordingly, larger spines tend to persist for long periods of time, whereas smaller, thinner spines are more transient [15, 17]. However, recent data suggests that these phenomena may be different between the cortex and hippocampus, with spines on CA1 hippocampal neurons demonstrating a more rapid turnover as compared to those found in cortical regions [18]. Nevertheless, many reports demonstrate that dendritic spines are not static structures and can rapidly reorganize in response to diverse stimuli including experience-dependent learning [19–21], as well as neuromodulatory and even hormonal signals [22–25]. One key sequela of this structural dynamism is the ability to sample the surrounding neuropil for incident axons [19, 26, 27].

**Figure 2:**
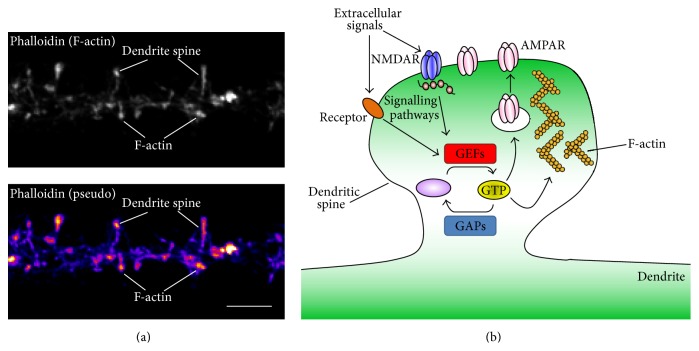
Dendritic spines, small GTPases, and the cytoskeleton. (a) Example of a cortical neuron immunostained with phalloidin, a marker of endogenous F-actin. Immunofluorescence reveals an enrichment of actin in dendrites and dendritic spines. (b) Schematic drawing of how extracellular signals can act via specific receptors and act via small GTPases to regulate actin dynamics and/or receptor trafficking. The dynamic actin cytoskeleton confers much of the structure of the dendritic spines, and alterations in synaptic expression of glutamate receptors (e.g., AMPA receptors) are thought to play a major role in modulating synaptic function.

It is widely recognized that dendritic spines are an integral component in circuit formation, but the precise nature of their contribution is still a topic of inquiry and debate. Dendritic spines exhibit a wide spectrum of structural reorganization, from formation and elimination, to more subtle changes in size and shape. These structures are estimated to contain over 1000 different proteins [28], including scaffolds, receptors, adhesion proteins, signalling proteins, F-actin, and cytoskeletal proteins (Figures 2(a) and 2(b)). Current theories postulate that dendritic spines provide a chemical and electrical signalling domain that is partially discrete from their parent dendrite, thus enhancing the computational capacity of the neuron [3], and that they are sufficiently enriched with the molecular components necessary for structural and function modifications [29]. Critically, the development, refinement, and maintenance of telencephalic neural circuits are essential for sensory perception, motor control, cognition, and memory [1, 8, 30, 31]. Importantly, a better understanding of circuit dynamics can provide a bridge between plasticity phenomena observed at the synapse and animal behaviour [8, 9, 18, 19]. Thus it is essential to examine mechanisms that rewire the brain and the current review is dedicated to this purpose. In the past decade, enormous progress has been made in dissecting the molecular mechanisms that contribute to the structural plasticity of dendritic spines [10, 12, 32, 33]. A key molecular determinant of dendritic spine plasticity is the actin cytoskeleton and its regulators. Here we review recent work that has begun to unravel the complex manner in which the family of small GTPases proteins, their regulators, and effectors modulate the actin cytoskeleton to control dendritic spine morphology in support of synaptic function.

## 2. Actin: A Key Determinant of Dendritic Spine Morphology

The morphological malleability of dendrite spines has been shown to be due to a dynamic actin cytoskeleton [34, 35]. Spines are rich repositories of filamentous and monomeric actin and achieve both stability and dynamism through a turnover process known as treadmilling, where monomers are simultaneously added to the barbed end (at the spine periphery) and removed from the pointed end of the filament (near the spine's core) [36, 37]. A variety of proteins exhibit control over the actin cytoskeleton and many of these proteins are potent spine morphogens and synaptic modulators [23, 38–42].

Tight control of the actin cytoskeleton is crucial to proper synaptic function. Indeed, actin treadmilling controls the distribution of proteins in the postsynaptic density, including AMPA receptors, as revealed by work employing fluorescence recovery after photobleaching [43]. Thus, understanding the complex signalling pathways impinging on actin filaments is critical for revealing mechanisms underlying normal and pathological synaptic transmission. To this end, much research effort has focused on identifying and characterizing actin regulatory proteins. By considering the positioning of these proteins in signalling cascades relative to the extracellular space and the actin cytoskeleton, they can be organized into hierarchical functional groups including actin binding proteins, small GTPases, and small GTPase regulators and effectors (Figure 2(b)) [32, 44].

## 3. Small GTPases: Morphological Signalling Hubs in Dendritic Spines

The super family of small GTPases is classified into 5 subfamilies: the Ras, Rho, Rab, Sar1/ARF, and Ran families. Members of this superfamily regulate diverse cellular functions and are often referred to as molecular switches as they exist in binary “on” and “off” states when bound to GTP and GDP, respectively [45, 46]. The present review will be limited to members of Rho and Ras families as these proteins have been most directly linked with actin remodelling. Further, Rho- and Ras-mediated signalling pathways exhibit substantial cross talk that has important implications for spine morphological and functional plasticity. While our understanding of small GTPase control of the actin cytoskeleton has been greatly enhanced by work in nonneuronal cells, the dendritic spine represents a unique microdomain, with distinct functional requirements. As such, we will focus on studies conducted in dendritic spines unless otherwise noted.

Extensive literature links the Rho subfamily to regulation of synaptic actin structure and dynamics [47]. Perhaps best studied among these family members are Rac1 and RhoA, which have potent and opposite effects on the structure of dendritic spines [48]. Overexpression of dominant negative Rac1 leads to reduced spine density in hippocampal slices and dissociated cultures [49, 50], while overexpression of a constitutively active form or RhoA leads to spine loss [51]. It is generally accepted that Rac1 activation stimulates F-actin polymerization and stabilizes dendritic spines through the activation of downstream effectors p21-activated kinase (PAK), LIM-kinase-I (LIMK-I), and the actin binding protein cofilin [52, 53]. Conversely, RhoA activation stimulates F-actin polymerization through its downstream protein kinase ROCK, which in turn directly regulates LIMK-1 phosphorylation in nonneuronal and neuronal cells [54, 55]. Rho GTPases are rapidly and locally activated in spine heads following potentiating stimuli as revealed by two-photon fluorescence lifetime imaging of FRET-based probes [55]. Interestingly, Cdc42, a Rac-related Rho GTPase, and RhoA exhibited differential spatial activity, reflecting their unique contributions to spine morphology regulation; blockade of the RhoA signalling cascade inhibited initial spine growth while Cdc42 pathway inhibition prevented sustained spine enlargement. Reinforcing the importance of Rho GTPases in forebrain plasticity is a recent study demonstrating active Rac1-induced spine proliferation in cortical pyramidal neurons as well as enhanced plasticity of visual circuits in monocularly deprived animals [56, 57]. In concordance with this idea, disruption of signalling through Rho/Rac pathways is frequently associated with intellectual disability (ID), a condition characterized by abnormalities in dendritic spine morphology [58–60].

Though most investigations of neuronal structure have focused on the Rho GTPase subfamily, other GTPases have been shown to regulate dendritic spine morphology. Members of the Ras subfamily of small GTPases have also been found to regulate dendritic spine structure and dynamics [61]. One of the first studies to link Ras with structural remodelling of dendritic spines was from a mouse model where a constitutive active form of H-Ras was overexpressed [62]. These mice displayed increased neuronal complexity, which was mirrored in subsequent studies which also revealed abnormal spine formation and connectivity [63, 64]. Consistent with a role in mediating dendritic spine plasticity, it has also been shown that Ras is activated concurrently with spine enlargement induced by uncaging of glutamate in hippocampal neurons [65]. Interestingly, the spatiotemporal dynamics of Ras activation was again different to that of the Rho GTPases, RhoA, and Cdc42, reinforcing the idea that both the temporal activation and the localization of these molecules are critical in determining their impact on cellular function [55, 65, 66]. Prior work in nonneuronal cells has also linked Rap, a member of the Ras subfamily, to cytoskeletal dynamics [67]. In neurons, activation of Rap1 by NMDA receptors in cultured cortical neurons results in a decrease in spine size [41]. Another powerful regulator of small GTPase activity in neuronal cell is the estrogen hormone, 17*β*-estradiol [68–70]. Interestingly, when mature cortical neurons are acutely exposed to 17*β*-estradiol, a rapid increase in active Rap1 is seen concurrent with an increase in spine density [25]. Critically, overexpression of RapGAP, a protein that inhibits Rap activation, blocked the effect of 17*β*-estradiol on spine density [25]. In contrast, overexpression of constitutively active Rap2 causes a loss of dendritic spine density and an increase in the number of filopodia-like protrusions in culture hippocampal neurons [71]. Consistent with these observations* in vitro*, mice that express a constitutively active Rap2 display fewer dendritic spines and impaired learning [72]. Collectively, these data demonstrate that Rho and Ras family GTPases have potent regulatory effects on dendritic spines which can impact cognitive function.

## 4. Small GTPase Regulators

GTPases are themselves tightly regulated by two classes of proteins: guanine nucleotide exchange factors (GEFs) which facilitate the binding of GTP by the GTPase and GTPase activating proteins (GAPs) which catalyze the hydrolysis of GTP to GDP. These proteins convey diverse signals from the extracellular space to GTPases and differ in their cellular expression patterns and intracellular distributions. Each GTPase can be regulated by a variety of different GEFs and GAPs, allowing for both signalling diversity and spatial specificity. Through catalyzing the exchange of the GTPase bound GDP to GTP, GEFs serve to activate GTPases. By responding to extracellular signals including neuromodulators and neuronal activity, GEFs can achieve bidirectional control over spine morphology and synaptic strength by acting through their target GTPases.

As RhoA is associated with spine shrinkage and destabilization, GEFs that activate this GTPase have similar effects on dendritic spine morphology. For example, GEF-H1 has been shown to colocalize with the AMPA receptor complex and negatively regulate spine density and length through a RhoA signalling cascade [73]. Similarly, activation of the Eph receptor A4 (EphA4) results in the retraction of dendritic spines, an effect that is dependent on activation of RhoA via its GEF, ephexin1 [74]. Another GEF involved in the destabilization and shrinkage of spines is Epac2. This multidomain Rap1 GEF is activated by cAMP and leads to reduced spine AMPA receptor content, depressed excitatory transmission, and spine destabilization as demonstrated by live imaging studies. Conversely, inhibition of Epac2 leads to spine enlargement and stabilization [23]. Interestingly, rare* de novo* mutations of the* Epac2* gene have been found to be associated with individuals with autism spectrum disorders (ASDs) [75]. The resulting mutant Epac2 proteins displayed altered abilities to activate Rap and when expressed in primary cortical neurons, they resulted in a range of abnormal dendritic spine morphologies [23]. Analysis of* Epac2* knockout mice has further revealed deficits in social and communicative behaviours, whereas memory and leaning behaviours are seemingly unaffected [76]. Interestingly, these mice also display reduced dendritic spine turnover* in vivo*, consistent with what has been shown previously* in vitro* [23, 76]. However, it is not clear how alterations in dendritic spine plasticity are linked with altered social and communicative behaviours. More recently, using* in utero* electroporation to express an RNAi construct against Epac2 in a subset of layer 2/3 cortical neurons, a role for Epac2 in maintenance of basal, but not apical, dendrites has been revealed [77]. Interestingly, regulation of basal dendrite formation by Epac2 requires Ras signalling, as a ASD-associated mutant Epac2 protein, which has a reduced ability to bind active Ras, also induces deficits in basal dendrite maintenance [77]. This demonstrates that there can be a level of cross talk between small GTPase systems. Consistent with this, it has recently been shown that the polo-like kinase 2 (Plk2) regulates both Ras and Rap activity through directly influencing the activity regulatory proteins of each small GTPase in response to homeostatic plasticity [78]. These studies demonstrate that the synchronized regulation of both Ras and Rap small GTPases via their GEFs and GAPs plays an important role in homeostatic plasticity and in the maintenance of neuronal morphology [77, 78].

The regulation of Rac by its GEFs has also been well studied. One such GEF is kalirin-7, which is especially unique due to the fact that it is the only known Rac1 GEF expressed in the cortex of adult mice [32]. Overexpression of this kalirin-7 in cortical cultures leads to an increase in spine head area and density. Concomitantly, knockdown of kalirin-7 through an RNAi approach reduces the spine area and density [42]. Interestingly, mice in which the* kalirin* gene has been deleted exhibit many phenotypes reminiscent of schizophrenia including deficits in working memory as well as reduced dendritic spine density in the cortex [79]. In the hippocampus, the role of kalirin-7 is obscured due to the presence of two other Rac1 GTPases, Tiam1 and *β*-PIX [32, 52, 80]. Tiam1 is regulated by NMDA receptor activation and has also been implicated in EphB receptor-dependent dendritic spine development [80, 81]. Likewise, the Rac1 GEF *β*-PIX, a downstream target of NMDA receptors, has been shown to be regulated by CaM kinase kinase and CaM kinase I [52].

Select GAPs have received research attention due to their putative roles in ID. Loss of the Rho-GAP oligophrenin-1, a gene implicated in ID, disrupts activity-dependent synapse and spine maturation [82]. Another such gene is the Ras-GAP SYNGAP1, which can regulate spine morphology through its target Ras as well as downstream signalling to Rac and cofilin [83]. This study illustrates that small GTPase signalling is often complex and nonlinear and may feature cross talk between pathways. Mutations in* SYNGAP1* have also been associated with both ID and ASD [84]. Interestingly, an animal model of human* SYNGAP1* haploinsufficiency displayed accelerated dendritic spine maturation resulting in disrupted excitatory/inhibitory balance in neural networks [85]. Moreover, these mice also developed persistent behavioural abnormalities. Critically, these effects were most prominent when SYNGAP1 was disrupted during early development and minimal when disrupted in adulthood [85]. More recently, SYNGAP1 has been shown to be phosphorylated by CaMKII, resulting in the trafficking of this protein away from synapses in response to LTP stimulation. Importantly, removal of this GAP protein from synapses is thought to be required for LTP-dependent Ras activation and subsequent AMPA receptor insertion and spine enlargement [86].

A number of extracellular signals are known to exert profound influences over dendritic spine morphology, through the activation of small GTPase pathways. The predominant receptor in regulating dendritic spine plasticity in response to synaptic activity is the NMDA receptor. Following activation of NMDA receptors, dendritic spines undergo a transient increase in calcium concentration [87, 88]. This rise in calcium activates the calcium-sensing calmodulin (CaM): calcium-bound CaM subsequently activates the CaMK family of serine/threonine kinases including CaMKI, CaMKII, and CaMKIV [89]. These kinases go on to phosphorylate a variety of targets involved in spine structural plasticity, including the Rac-GEF kalirin-7, as well as other signalling and scaffolding proteins involved in plasticity [42, 90]. Aside from glutamate, other neurotransmitters have been shown to modulate dendritic spine plasticity. Activation of 5-HT2A receptors in pyramidal neurons increased spine size through a kalirin-7-Rac1-PAK-dependent mechanism [22]. This study is of particular importance as it provides a direct link between serotonergic signalling and dendritic spine morphogenesis, both implicated in schizophrenia. Another important neurotransmitter implicated in the modulation of dendritic spines and small GTPase function is dopamine [91]. For example, treatment of rats with 6-hydroxydopamine, a neurotoxin that selectively ablates dopaminergic and noradrenergic neurons, resulted in a decrease in dendritic spine density in the prelimbic cortex 3 weeks after toxin administration [92]. Intriguingly, cognitive deficits in schizophrenia have been linked with dopamine dysfunction [93, 94] and reduced dendritic spine density has been observed in postmortem tissue taken from schizophrenic patients [95–97]. Results from Solis et al. suggest that there may indeed be a pathological link between dopamine dysfunction and loss of dendritic spine density. A finding consistent with this idea is that treatment with the atypical antipsychotic olanzapine, but not the typical antipsychotic haloperidol, was able to rescue 6-hydroxydopamine-induced spine loss in the rat prefrontal cortex [98]. At the molecular level, activation of the D1/D5 receptors with the selective agonist SKF-38393 leads to spine shrinkage through activation of the Rap GEF Epac2 [23].

Less conventional neuromodulators have also been implicated in the regulation of dendritic spines. Classically defined as a hormone, estrogens have recently come into the spotlight as an important modulator of dendritic spine plasticity [99]. Treatment of primary cortical cultures with 17*β*-estradiol increased spine density while decreasing the AMPA receptor content of spines. These “silent synapses” were potentiated by activation of NMDA receptors, reminiscent of activity-dependent maturation of silent synapses during development [25]. These effects were mediated by the Rap/AF-6(afadin)/ERK1/2 signalling pathways, as inhibiting or interfering with the actions of these proteins was sufficient to block 17*β*-estradiol's effects on spines [25]. Additionally, recent studies have demonstrated that acute treatment of rat cortical cultures with 17*β*-estradiol leads to phosphorylation of WAVE1 and its subsequent targeting to spines, resulting in the polymerization of actin. This is thought to be required for the formation of immature dendritic protrusions in young cortical neurons [100]. Similar findings have been reported in hippocampal cultured neurons. Here, chronic treatment of hippocampal cultures with 17*β*-estradiol resulted in an increased number of synapses and increased localization of kalirin-7 to dendritic spines [101]. However, these actions of 17*β*-estradiol seem to be mediated through the estrogen receptor beta (ER*β*) as activation of ER*β* but not ER*α* agonists is able to recapitulate these effects [101–104].

## 5. Small GTPase Effectors and Actin Binding Proteins

Downstream of small GTPases is a series of effector proteins which convey signals to direct regulators of the actin cytoskeleton. A particularly well-described family of effectors of the Rho GTPases Rac1 and Cdc42 are the p21-activated kinases (PAKs) [105] and the Rho kinases (ROCK) [106]. The PAKs are critical for spine morphogenesis and synaptic structure, particularly in the cortex [107]. More recently, a series of studies has explored the consequences of PAK and ROCK knockout in the forebrain. Deletion of PAK1 or ROCK-2 results in the loss of F-actin from spines [108, 109]. Further, both knockout animals demonstrated deficits in hippocampal LTP, highlighting the importance of these Rho kinases for synaptic plasticity. Intriguingly, codeletion of PAK1 and PAK3 resulted in a more severe structural and functional phenotype; the PAK1/3 knockouts showed impaired bidirectional plasticity in the hippocampus, deficits in learning and memory, and gross structural abnormalities in the forebrain [110]. Shared features of these Rho kinase knockout animals include disruption of the kinase cascade downstream of the Rho GTPases, a release of cofilin from inhibition, and a subsequent loss of F-actin from dendritic spines.

More insight into the effects of PAK and ROCK family members on the actin cytoskeleton is provided by work examining LIM-kinase (LIMK). Active Pak1 can phosphorylate LIMK-1 which in turn inhibits cofilin activity [111]. As a result, genetic ablation of LIMK-1 results in elevated cofilin activity, aberrant spine morphology, and enhanced LTP [53]. Intriguingly, recent work has identified a new mechanism of regulation for LIMK-1 via lipid modification [24]. N-terminal palmitoylation of LIMK-1 targets the kinase to dendritic spines and is necessary for activity-dependent spine growth. Palmitoylation is emerging as a critical modulator of spiny synapse function [112]; small GTPases themselves are targeted to various microdomains through dynamic palmitoylation [113–115], though the implications of this signalling have yet to be explored thoroughly in neurons.

As their name suggests, actin binding proteins directly influence actin dynamics through nucleating, stabilizing, or severing actin filaments. Members of the Wiskott-Aldrich syndrome protein (WASP) family bind both monomeric and filamentous actin [116] and are relieved from autoinhibition by Rho GTPases [117]. N-WASP, a brain enriched WASP, appears to be critical for spine and excitatory synapse formation [40]. Small GTPases also exert control over a similar WASP-family verprolin-homologous protein (WAVE) family. These proteins play a role in spine maintenance [118] and formation [119]; deficient WAVE1 expression is accompanied by spatial memory deficits in mice [120].

The Arp2/Arp3 complex is a well-studied actin nucleator and facilitator of actin branching [121]. The Arp2/Arp3 complex is downstream of Rho family GTPases, WASP, and WAVE proteins [122] and is likely to be instrumental in dendritic spine remodelling during spine growth [123]. Inhibition of the Arp2/Arp3 complex by protein kinase C binding protein (PICK1) is necessary for spine shrinkage during LTD [124]. More recently, PICK1 has been shown to signal downstream of AMPARs to inactivate Cdc42 [125]. As mentioned above, cofilin is another critical determinant of actin skeletal dynamics and competes with the Arp2/Arp3 complex by severing and debranching actin filaments [126]. Though prolonged cofilin activation promotes a reduction in spine size [127], it appears that a transient burst of cofilin activity is required for spine growth during chemically induced LTP [128]. A recent review of small GTPase control of the actin cytoskeleton covers these pathways in greater detail [44].

Among the list of Rap effectors are a number of actin cytoskeleton regulators. Rap1 binds directly to afadin, also known as AF-6 [129] which is a multidomain scaffolding protein instrumental in cell-cell adhesion [130]. Indeed, active Rap was responsible for the subcellular targeting of afadin in neurons under basal and after NMDA receptor activation [41, 131]. Intriguingly, following activation of NMDA receptors, afadin translocates to both synapses and the nucleus in a time-dependent manner. At synapses, afadin is required for activity-dependent and Rap-dependent spine modifications [41], whereas in the nucleus, afadin is required for the time-dependent phosphorylation of H3 histones, suggesting a potential role in regulating activity-dependent gene transcription [131]. Afadin also directly interacts with the actin-polymerizing protein profilin [129] and with the adhesion protein, N-cadherin [132], and the AMPA receptor subunit, GluA2 [133]. Consistent with these interactions, afadin is required for linking N-cadherin with the kalirin-7, therefore allowing regulation of Rac activation and linking N-cadherin with the dynamic modulation of dendritic spine morphology [132]. Moreover, knockdown of afadin using an RNAi approach results in a loss of dendritic architecture, dendritic spine density, and AMPA receptor mediated transmission [133]. Rap has also been shown to interact with and activate the Rac-GEFs Vav2 and Tiam1 [134], providing another example of small GTPase pathway cross talk.

Thus, a stereotyped spine-morphogenic signalling cascade begins with an extracellular signal that is conveyed to GEFs or GAPs that control small GTPase activity, which in turn influences actin binding proteins through small GTPase effectors. It is now emerging that, in addition to activity-dependent signalling via NMDA receptors, other extracellular signals, including neuromodulators [22, 23] and neurosteroids, may act via similar pathways.

## 6. Conclusions

Understanding how neurons encode information is a fundamental challenge in determining how we store and retrieve information about our surrounds, allowing us to adapt at a behavioural level. Growing evidence indicates that a key cellular correlate of information encoding is the regulation of dendritic spines and thus excitatory synaptic connections [1, 3]. In this review, we have presented recent evidence that places small GTPase proteins as an important intermediate between extracellular signals and the actin cytoskeleton, allowing for the regulation of synapse structure and function. Important advances have been made in our understanding of the molecules that exert a tight regulation of small GTPase function in neurons [32, 61], and it is also emerging that these molecules have unique spatiotemporal dynamics that are critical to their cellular functions [55, 65, 66]. Our current understanding suggests that small GTPases can act independently, via their effectors, directly regulating the actin cytoskeleton, to exert effects of dendritic spine structure and numbers, as well as on synaptic function. However, several studies have now demonstrated that multiple small GTPases can act in cooperation to bring about changes in dendritic spine, or on the maintenance of overall neuronal morphology [77, 78]. Moreover, it is also emerging that a wide range of extracellular signals also signal via small GTPases to exert morphogenic actions [22, 25, 42, 47, 50, 65, 74, 80, 81]. Many of these extracellular signals can activate the same small GTPases, suggesting that within a single neuron multiple factors can modulate the activity of a single subfamily of small GTPase. Elucidating how neurons integrate multiple signals and how they in turn summate impacting the function of the cell and ultimately affect cognition is fast emerging as another challenge. It is likely that gaining a greater understanding of the spatiotemporal dynamics of small GTPase signalling will provide an insight into how neurons handle this amount of information. In addition, further determining the complex manner in which regulators of small GTPase signalling interact and determining the nonlinear manner in which multiple pathways are activated by the same signals will provide a more comprehensive understanding of how multiple factors regulate spine plasticity.

It is also of note that multiple neurodevelopmental, psychiatric, and neurodegenerative disorders have been strongly associated with disruptions of neural circuits [6, 135]. Indeed, numerous neuropathological postmortem studies have strongly linked abnormal spine morphology with the pathogenesis of a number of neuropsychiatric, neurodevelopmental, and neurodegenerative disorders [135, 136], such as ID [137], fragile-X [138], Down's syndrome [139], autism spectrum disorders (ASDs) [140–142], schizophrenia [96, 143], depression [144], and Alzheimer's disease [145, 146]. It is currently posited that dendritic spine dysmorphogenesis can lead to defective or excessive synapse function and connectivity, resulting in disruptions in neural circuitry. This topic has recently been reviewed in depth [2, 6, 135]. Dysregulation of the complex mechanisms that control dendritic spine structure and function may contribute to these synaptic irregularities. Understanding the cellular mechanisms by which dendritic spine morphogenesis occurs will expand not only our knowledge of normal brain function, but that of abnormal brain function as well. Though a greater understanding of the cellular mechanisms that underpin cortical plasticity will be required, harnessing structural plasticity may offer a powerful future therapeutic avenue for neuropathologies.
